# Immediate weight-bearing is safe following lateral locked plate fixation of periprosthetic distal femoral fractures

**DOI:** 10.1186/s43019-021-00097-0

**Published:** 2021-06-25

**Authors:** Oisin J. F. Keenan, Lauren A. Ross, Matthew Magill, Matthew Moran, Chloe E. H. Scott

**Affiliations:** Edinburgh Orthopaedics, Royal Infirmary of Edinburgh, 51 Little France Crescent, Edinburgh, EH16 4SA UK

**Keywords:** Periprosthetic fracture, Distal femur fracture, Lateral locking plate fixation, Weight-bearing

## Abstract

**Purpose:**

This study aimed to determine whether unrestricted weight-bearing as tolerated (WBAT) following lateral locking plate (LLP) fixation of periprosthetic distal femoral fractures (PDFFs) is associated with increased failure and reoperation, compared with restricted weight-bearing (RWB).

**Materials and methods:**

In a retrospective cohort study of consecutive patients with unilateral PDFFs undergoing LLP fixation, patients prescribed WBAT were compared with those prescribed 6 weeks of RWB. The primary outcome measure was reoperation. Kaplan–Meier and Cox multivariable analyses were performed.

**Results:**

There were 43 patients (mean age 80.9 ± 11.7 years, body mass index 26.8 ± 5.7 kg/m^2^ and 86.0% female): 28 WBAT and 15 RWB. There were more interprosthetic fractures in the RWB group (*p* = 0.040). Mean follow-up was 3.8 years (range 1.0–10.4). Eight patients (18.6%) underwent reoperation. Kaplan–Meier analysis demonstrated no difference in 2-year survival between WBAT (80.6%, 95% CI 65.3–95.9) and RWB (83.3%, 95% CI 62.1–100.0; *p* = 0.54). Cox analysis showed increased reoperation risk with medial comminution (hazard ratio 10.7, 95% CI 1.5–80; *p* = 0.020) and decreased risk with anatomic reduction (hazard ratio 0.11, 95% CI 0.01–1.0; *p* = 0.046). Immediate weight-bearing did not significantly affect the risk of reoperation compared with RWB (relative risk 1.03, 95% CI 0.61–1.74; *p* = 0.91).

**Conclusions:**

LLP fixation failure was associated with medial comminution and non-anatomic reductions, not with postoperative weight-bearing. Medial comminution should be managed with additional fixation. Weight-bearing restrictions additional to this appear unnecessary and should be avoided.

## Introduction

The number of primary total knee arthroplasties (TKAs) performed is increasing annually [1]. In the context of an aging population [2], periprosthetic distal femoral fractures (PDFFs) represent an increasing burden on patients and surgeons [3–5], with a current incidence estimated at 2.4 per 100,000 population per year [6]. These fractures are typically fragility fractures affecting older and often frail patients [7]. Where fractures are proximal to well-fixed femoral components with adequate bone stock, they are usually managed with fixation, as opposed to revision arthroplasty [4]. Although proximal diaphyseal-type fractures can be treated with retrograde femoral nailing [8], where fixation is undertaken for more distal fractures a lateral locking plate (LLP) is typically used, with or without additional augmentation [9, 10].

Guidelines for the management of femoral fragility fractures emphasize the importance of early mobilization [11, 12]. Unrestricted early weight-bearing improves functional mobility and the likelihood of discharge home, and reduces both complications and mortality in these vulnerable patients [13–15]. These guidelines are generally followed for fractures of the proximal femur [16]; however, similar arguments are not always made for fractures of the distal femur, for which weight-bearing restrictions are often prescribed due to concerns regarding fixation failure [9, 17–20]. This has led some authors to recommend distal femoral replacement in preference to fixation, in order to facilitate immediate and unrestricted weight-bearing [21, 22]. It has previously been shown in two cohorts of patients with distal femoral fractures, including both native and periprosthetic fractures, that modern locking plates facilitate safe early weight-bearing with low rates of failure in the management of distal femoral fractures [5, 23]. In studies limited to patients with PDFFs, postoperative weight-bearing status has either been restricted [9, 20] or not reported [10]. The safety of unrestricted weight-bearing after LLP fixation of PDFFs has been reported by Smith et al. [24], although no comparative group was included.

The aim of this retrospective study was to determine whether immediate unrestricted weight-bearing following LLP fixation of PDFFs was associated with increased fixation failure and reoperation, compared with 6 weeks of restricted weight-bearing (RWB). The null hypothesis was that there would be no difference in failure and reoperation rates between the unrestricted and RWB groups. Secondary outcomes included perioperative complications, functional mobility status, length of acute hospital stay, discharge destination and mortality.

## Materials and methods

This retrospective cohort study was approved by the institutional musculoskeletal audit and quality improvement group. Consecutive patients with PDFFs involving well-fixed TKAs treated at the study institution between January 2011 and December 2019 were identified from admission lists and operating lists. Patients with bilateral simultaneous fractures, the second of bilateral sequential fractures, intraoperative periprosthetic fractures and fractures that were not treated with LLP fixation were excluded. All patients in the study population underwent internal fixation with an LLP +/− augmentation, performed by one of eight specialist orthopaedic trauma surgeons.

A PDFF was defined as suitable for fixation by the operating surgeon when the TKA femoral component was well fixed with sufficient bone in the distal fragment to receive five locking screws through an LLP. A Periloc (Smith & Nephew, Watford) LLP was placed using a minimally invasive, direct lateral or lateral parapatellar approach. Additional fixation with cables, lag screws or dual plating was performed at the surgeon’s discretion, according to fracture configuration. Locking screws were used distally and non-locking screws were used proximally, typically via the targeting device. Postoperative weight-bearing restrictions were prescribed at the discretion of the operating surgeon. Patients were prescribed either immediate and unrestricted weight-bearing as tolerated (WBAT) or 6 weeks of RWB, which included instructions for either partial, toe-touch or non-weight-bearing. Patients were mobilized from the first postoperative day or when medically fit. Mobilization proceeded from hoist to gutter frame to zimmer frame to walking sticks or crutches, according to patient ability, under the supervision of physiotherapists, occupational therapists and nurses. Patients in the RWB group did not progress beyond their prescribed restriction. Patients in the WBAT group progressed as their symptoms and ability allowed.

Electronic patient records and operation notes were examined and the following data recorded: demographic data, body mass index (BMI), date of primary prosthesis, date of injury, osteoporosis, bisphosphonate use, details of operative management, weight-bearing restrictions and complications (early < 6 weeks and late > 6 weeks). Details of the surgical approach and fixation construct were recorded (plate type and configuration, plate length, working length, cable use and proximal screw number). Functional mobility status was recorded pre fracture and at discharge. A score was assigned from 0 (full independent activity) to 5 (bedridden) [25]. The acute length of stay and discharge destination were determined. Clinical follow up was performed at 6–8 weeks. Further clinical follow up was at the surgeon’s discretion. Mortality was calculated at 30 days, 90 days and 1 year. Modes of surgical management failure were determined and details of reoperation were recorded.

Radiographic review was performed independently by two orthopaedic surgeons (CEHS and OJFK), using the picture archiving and communication system (PACS; Kodak Carestream, Rochester, NY, USA). Fractures were classified using the Su classification [26]. Interprosthetic fractures were noted. The fixation construct was recorded (LLP length, dual plates, lag screws, cerclage cables and proximal screw number). The presence of medial comminution and the quality of reduction were recorded and any malreduction deformity was noted. Anatomic reduction was defined as anatomic restoration of alignment and translation in coronal and sagittal planes. All subsequent radiographs were reviewed in the national PACS archive to identify any subsequent fixation failure or revision surgery that may have occurred outside our institution but within Scotland.

### Statistical analysis

Data were analysed using SPSS version 25.0. Univariate analysis was performed using parametric (unpaired Student’s *t* test) and non-parametric (Mann–Whitney *U* test) tests as appropriate to assess continuous variables for significant differences between the RWB and WBAT groups. Nominal categorical variables, such as revision and reoperation, were assessed using the chi-squared or Fisher’s exact test. *p* ≤ 0.05 was considered statistically significant. Kaplan–Meier analysis survival analysis was undertaken, using the end point of reoperation for any reason. The log-rank statistic was used to compare the two weight-bearing strategies. Cox multivariable regression analysis was performed to identify risk factors for reoperation, using the following covariates: age at fracture, postoperative weight-bearing restriction, Su classification, medial comminution, anatomic reduction obtained, residual golf-club deformity, dual plating and cable use. A power calculation suggested that a sample size of 42 would detect a 3.8× increase in reoperation rate from a baseline of 10% [10, 20], as significant with 80% power and *α* = 0.05.

## Results

From January 2011 to December 2019, 47 PDFFs involving TKAs occurred in 43 patients and were treated with LLP fixation. The second fractures of bilateral sequential fractures were excluded, leaving a study population of 43 PDFFs in 43 patients. The fractures occurred at a mean of 9.5 years (SD 3.6, range 0–21) after primary TKA, which incorporated a non-stemmed primary femoral component in 41 cases (95.3%) and a hinged knee prosthesis with femoral and tibial stems in 2 cases (4.6%). Seven (16.2%) fractures were interprosthetic. An LLP was used in isolation in 22/43 patients (51.2%) and was augmented by cables in 13/43 (30.2%), by lag screws in 3/43 (7.0%) and by a medial plate (dual plating) in 5/43 (11.6%). In 14/43 cases (32.6%), a minimally invasive approach was used. The mean length of follow-up was 3.6 years (SD 2.8, range 1.0–9.1).

### Weight-bearing status

Postoperatively, immediate WBAT was prescribed in 28/43 (65.1%) patients and RWB in 15/43 (34.9%). The patients’ demographic and clinical characteristics are detailed in Table 1. These did not differ between the WBAT and RWB groups, other than a higher frequency of patients with interprosthetic fractures in the RWB group: 5/15 (33.3%) RWB patients had interprosthetic fractures, compared with 2/28 (7.1%) in the WBAT group (*p* = 0.040, Fisher’s exact test). RWB was prescribed for 6 weeks and included: partial weight-bearing in 5/15 patients; touch weight-bearing in 3/15 patients; and no weight-bearing in 7/15 patients. A hinged knee brace with unrestricted flexion was used in 4/28 patients (14.3%) in the WBAT group and in 1/15 patients (6.7%) in the RWB group.

**Table 1:** Patient and fracture characteristics

Variable	WBAT (***n*** = 28)	RWB (***n*** = 15)	***p*** value
Age (years)	83.1 (11.6)	76.8 (11.1)	0.092*
BMI	26.5 (5.6)	27.6 (6.1)	0.591*
BMI ≥30	8 [28.6]	5 [33.3]	0.564^
Female gender	24 [85.7]	13 [86.7]	0.932^
Osteoporosis	9 [32.1]	2 [13.3]	0.273^^
Bisphosphonates	4 [14.2]	1 [6.7]	0.639^^
Pre-fracture functional mobility scale			
0 - Full activity	9 [32.1]	6 [40.0]	0.771^
1 - Walking with assistance	8 [28.6]	5 [33.3]	
2 - Walking with assistance for short periods only	4 [14.3]	3 [20.0]	
3 - Walking with assistance for ADLs/appointments only	2 [7.1]	0	
4 - Confined to a wheelchair	1 [3.6]	0	
5 - Bedridden	1 [3.6]	0	
**Fracture features**			
Su classification			0.891
I	6 [21.4]	4 [26.7]	
II	17 [60.7]	8 [53.3]	
III	5 [17.9]	3 [20.0]	
Medial comminution	4 [14.3]	5 [33.3]	0.238^^
Interprosthetic	2 [7.1]	5 [33.3]	0.040^
Time since TKA (years)	10.4 (5.7)	7.6 (4.5)	0.124*
**Surgical parameters**			
Time to surgery (days)	3	2	0.238**
Anatomic reduction	19 [67.9]	9 [60.0]	0.606^
Golf-club deformity	3 [10.7]	2 [13.3]	1.00^^
Cables	8 [28.6]	7 [46.7]	0.235^
Dual plating	3 [10.7]	2 [13.3]	1.00^^
Open approach	13 [46.4]	9 [60.0]	0.755^

Data presented as mean (SD) or number [%]

*BMI* body mass index, *RWB* restricted weight-bearing, *TKA* total knee arthroplasty, *WBAT* weight-bearing as tolerated

^*^ Unpaired T-test

^**^ Mann Whitney U test

^^^ Chi square

^^^^ Fisher's exact

### Primary outcome measure: reoperations

**Fig. 1:**
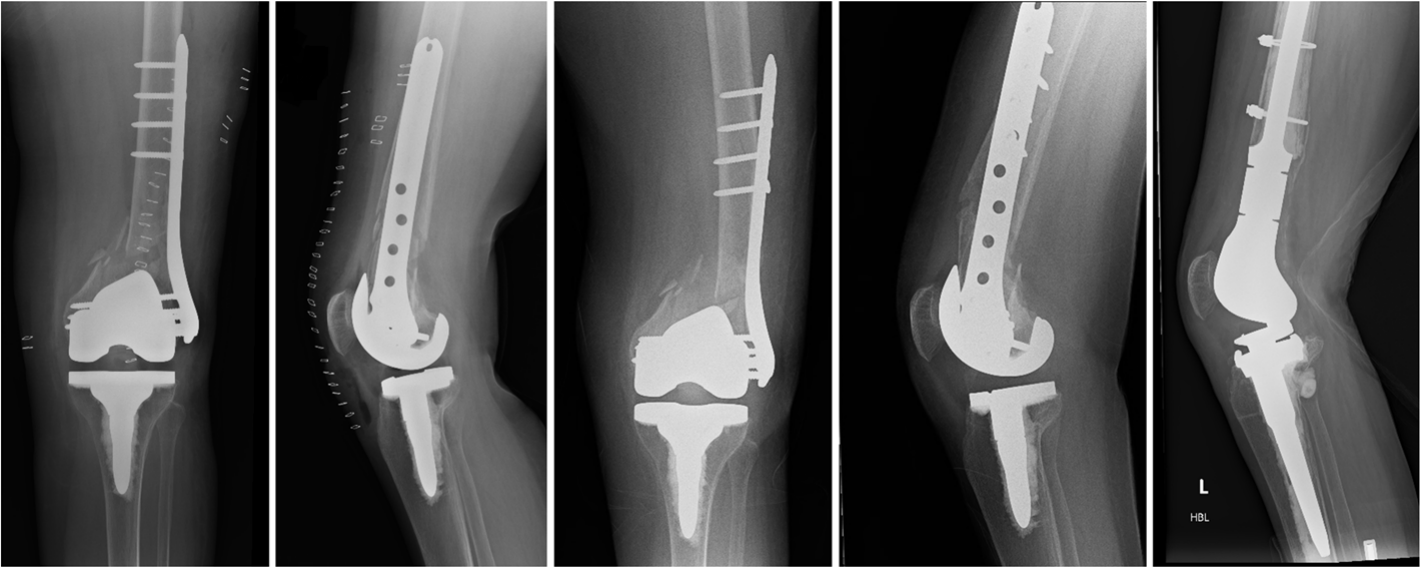
Early fixation failure at 6 weeks following lateral locking plate (LLP) fixation via a lateral parapatellar approach and weight-bearing as tolerated (WBAT) in a 75-year-old woman with a Su III periprosthetic distal femoral fracture with medial comminution and coronal plane malreduction (golf-club deformity). This was revised to a distal femoral endoprosthesis

**Fig. 2:**
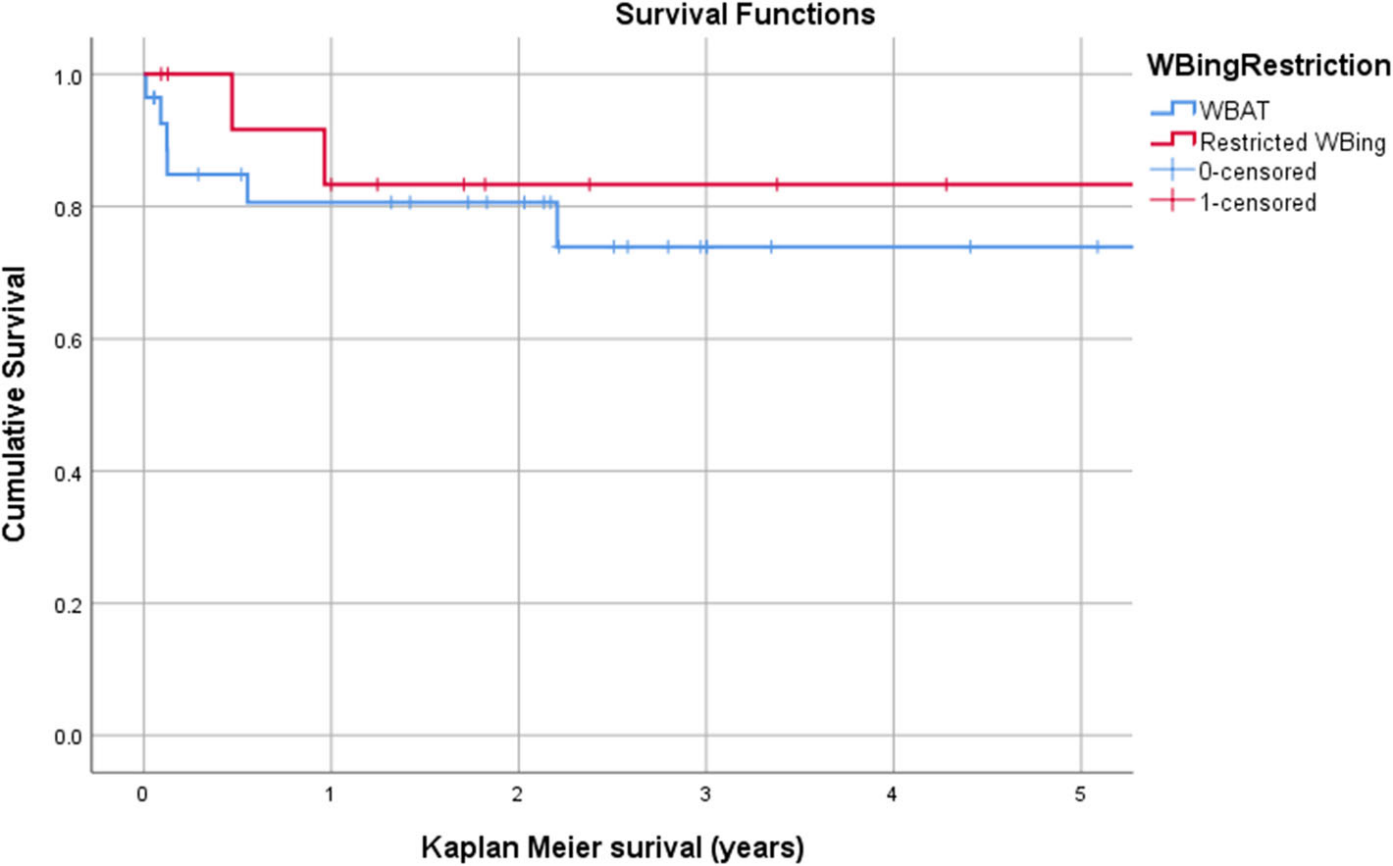
Kaplan–Meier survival analysis with the end point of reoperation, comparing those allowed weight-bearing as tolerated (WBAT) and those with restricted weight-bearing (WBing). No difference was identified up to 5 years (*p* = 0.542, log-rank test)

During the study period, eight patients (18.6%) underwent reoperation for any reason. This did not differ significantly between the WBAT (6/28) and RWB (2/15) groups (*p* = 0.69, Fisher’s exact test). The indications for reoperation are presented in Table 2, along with the fixation construct employed and the assumed mode of failure (Fig. 1). The relative risk of reoperation in the WBAT group, compared with the RWB group, was 1.03 (95% CI 0.61–1.74; *p* = 0.91). Kaplan–Meier analysis (with the end point of reoperation for any reason) demonstrated no difference in survival between the WBAT and RWB groups at 2 years: 80.6% (95% CI 65.3–95.9) following WBAT, compared with 83.3% (95% CI 62.1–100) following RWB (*p* = 0.54, log-rank test) (Fig. 2). Brace use was not associated with the reoperation rate: 1/5 brace users required reoperation (*p* = 1.0, Fisher’s exact test).

**Table 2:** Details of reoperations for the WBAT (*n* = 6) and RWB (*n* = 2) groups

Age (years)	Sex	Fracture features	Construct	Reduction	MoF	Cause	Mx
**WBAT**							
68	Female	Su III Medial comminution	LLP 13 hole; 5 proximal screws	Extended	Refracture	New fracture at proximal plate tip	DFR
87	Female	Su II	LLP 8 hole; cables; 4 proximal screws	Anatomic	NU and plate fracture	Excessive soft tissue stripping	Refix (IMN)
75	Female	Su III Medial comminution	LLP 13 hole; 4 proximal screws	Golf-club deformity	Early fixation failure	Coronal plane malreduction, medial comminution	DFR
79	Female	Su I	LLP 8 hole; 3 proximal screws	Anatomic	Early fixation failure	Inadequate fixation	Refix (LLP)
83	Female	Su II Medial comminution	LLP and cables	Slight varus	NU and plate fracture	Medial comminution	Refix (dual plating)
86	Female	Su II	LLP 8 hole; 4 proximal screws	Extended	Infection	Deep infection, tibial collapse	Revision TKA (Total Stabiliser)
**RWB**							
71	Female	Su III Interprosthetic Medial comminution	LLP 13 hole; lag screws; 6 proximal screws	Golf club, varus	NU and Plate fracture	Coronal plane malreduction Mixed modes of fixation	DFR
75	Female	Su II	LLP 13 hole; lag screws; 5 proximal screws	Anatomic	NU and plate fracture	Mixed modes of fixation	Refix (dual plating)

0 - Full mobility

1 - Walking with assistance

2 - Walking with assistance for short periods only

3 - Walking with assistance for ADLs/appointments onl

4 - Confined to wheelchair

5 - Bedridden

### Secondary outcome measures

**Fig. 3:**
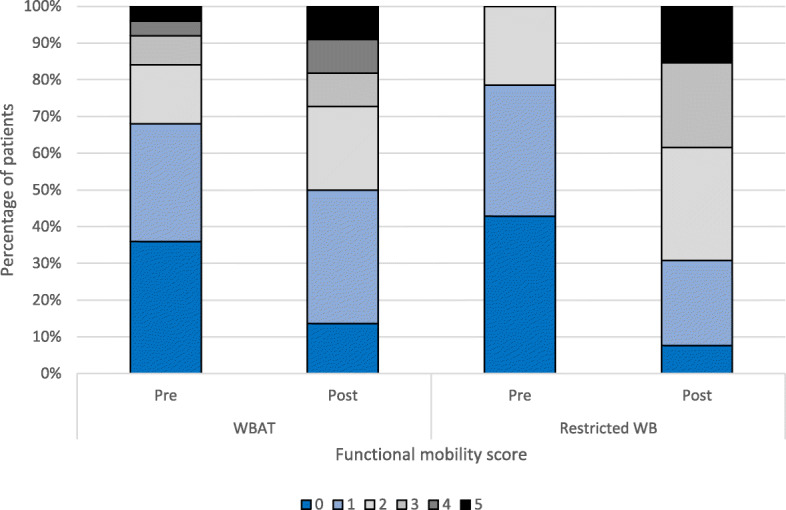
Functional mobility status before and after fixation of periprosthetic distal femoral fracture (PDFF) with a lateral locking plate (LLP) by postoperative weight-bearing (WB) restriction. WBAT, weight-bearing as tolerated

Complications and mortality rates are presented in Tables 3 and 4. Ten (23.1%) patients died during follow-up. There were no significant differences in complications, length of acute hospital stay, requirement for rehabilitation, discharge to home and ultimate functional mobility status between groups (Tables 3 and 4). Functional mobility status declined less in the WBAT group, but this was not significant (Table 3, Fig. 3).

**Table 3:** Cox logistic regression analysis (*p* = 0.026) to identify risk factors for reoperation following LLP

Covariate	Hazard ratio (95% CI)	***p*** value
Age	1.01 (0.90–1.12)	0.912
Su III fracture	5.58 (0.72–43.3)	0.100
Medial comminution	**10.7 (1.45–79.5)**	**0.020**
Anatomic reduction	**0.11 (0.013–0.96)**	**0.046**
Golf-club deformity	0.28 (0.03–2.59)	0.264
Dual plating	5.3 (0.12–224.1)	0.387
Cables	0.80 (0.14–4.50)	0.800
Protected weight-bearing postoperatively	0.33 (0.05–2.37)	0.269

*LLP* lateral locking plate

Bold data represent *p*-value of less than 0.05

**Table 4:** Postoperative complications following LLP fixation of PDFFs in patients allowed WBAT compared with those with RWB

Variable	WBAT (***n*** = 28)	RWB (***n*** = 15)	***p*** value
**Complications**			
Early medical (<6 weeks)	17 [60.7]	9 [60.0]	0.850^
Early surgical (<6 weeks)	5 [17.9]	0	0.076^
**Mortality**			
30 days	2 [7.1]	0	0.535^^
90 days	2 [7.1]	2 [13.3]	0.602^^
1 year	4 [14.2]	2 [13.3]	1.0^^
**Failures**			
Reoperations	6 [21.4]	2 [13.3]	0.692^^
Non-union	2 [7.1]	2 [13.3]	1.0^^
Fixation failure	2 [7.1]	0	0.535^^
**Mobility**			
Post-fracture FMS 0	3 [10.7]	1 [6.7]	0.623^
1 - Full activity	8 [28.6]	3 [20.0]	
2 - Walking with assistance for short periods only	5 [17.9]	4 [26.7]	
3 - Walking with assistance for ADLs/appointments only	2 [7.1]	3 [20.0]	
4 - Confined to wheelchair	2 [7.1]	0	
5 - Bedridden	2 [7.1]	2 [20.0]	
Functional mobility change	−0.67 (1.3)	−1.46 (1.3)	0.083*
**Disposition**			
Length of acute stay	13	10	0.265**
Required period of rehabilitation	12	8	0.651^
Ultimate discharge destination			0.841^
Own home	10 [35.7]	6 [40.0]	
Care home	4 [14.3]	1 [6.7]	
Community hospital	2 [7.1]	0	
Died in hospital	1 [3.6]	1 [6.7]	
Unknown after rehabilitation	10 [35.7]	6 [40.0]	

Data presented as number [%]

*LLP* lateral locking plate, *PDFF* periprosthetic distal femoral fracture, *RWB* restricted weight-bearing, *WBAT* weight-bearing as tolerated

^*^ Unpaired T-test

^**^ Mann Whitney U test

^^^ Chi square

^^^^ Fisher's exact

### Regression analysis

Cox multivariable logistic regression analysis was performed for the end point of reoperation for any reason (Table 5). Patient weight and BMI were not included as these data were missing for six patients and resulted in a model of insufficient significance (*p* = 0.24). Cox multivariable logistic regression analysis (*p* = 0.026) identified medial comminution as an independent predictor of reoperation following plate fixation of PDFFs (hazard ratio 10.7, 95% CI 1.5–80; *p* = 0.020) (Fig. 1). Anatomic reduction was an independent predictor of decreased risk of reoperation (hazard ratio 0.11, 95% CI 0.013–0.96; *p* = 0.046). Postoperative weight-bearing restrictions were not significantly associated with altered risk of reoperation (hazard ratio 0.33, 95% CI 0.05–2.4; *p* = 0.27).

**Table 5:** Details of medical and surgical complications

Complication	WBAT (***n*** = 28)	RWB (***n*** = 15)	***p*** value
**Early surgical**			0.076^a^
Another fracture	1 [3.6]	0	
Cellulitis	1 [3.6]	0	
Deep infection	1 [3.6]	0	
Fixation failure	2 [7.1]	0	
**Early medical**			0.850^a^
AF	0	1 [6.7]	
AKI	2 [7.1]	1 [6.7]	
Anaemia	1 [3.6]	2 [13.3]	
Electrolyte abnormality	1 [3.6]	0	
MI	2 [7.1]	0	
Pneumonia	6 [21.4]	0	
Sepsis	1 [3.6]	0	
UTI	2 [7.1]	4 [26.7]	
VTE	0	1 [6.7]	
Wound infection	1 [3.6]	0	
**Late surgical**			0.576^a^
Another fracture	1 [3.6]	1 [6.7]	
Aseptic loosening	1 [3.6]	0	
Deep infection	0	1 [6.7]	
Fixation failure	2 [7.1]	0	
NU and plate fracture	2 [7.1]	2 [13.3]	
Wound infection	0	1 [6.7]	

Data presented as number [%]

*AF* atrial fibrillation, *AKI* acute kidney injury, *MI* myocardial infarction, *NU* non-union, *RWB* restricted weight-bearing, *UTI* urinary tract infection, *VTE* venous thromboembolism, *WBAT* weight-bearing as tolerated

^a^Chi-square test

## Discussion

In the current study, immediate unrestricted weight-bearing following LLP fixation of PDFFs was not associated with an increased risk of fixation failure or reoperation compared with 6 weeks of RWB. The presence of medial comminution was identified as an independent risk factor for reoperation and anatomic reduction was associated with a reduced risk. Of eight reoperations, two were for early fixation failure in the WBAT group and both were attributable to other mechanical failures: coronal plane malreduction with medial comminution in one case; and insufficient proximal fixation (three proximal bicortical screws) in the other. RWB does not appear necessary in these frail older patients, when an LLP plate is used, provided an anatomic reduction is obtained and held with sufficient fixation. We recommend adequate proximal fixation be obtained in all cases with five bicortical screws [27] and that medial comminution is augmented with additional fixation.

A number of studies have investigated the role of LLP fixation in the management of PDFFs [10, 17, 18, 20, 28–30]. Over the past decade, surgeons have gained experience with the LLP technique and its augmentation, and surgical outcomes have generally improved: non-union rates have fallen from 24% [18] to approximately 10% in published series [10, 20]. Although some centres are increasingly prescribing unrestricted weight-bearing in older patients after extra-articular native distal femoral fractures [5, 23], this has not been the case in modern published series of PDFFs, where all have imposed some weight-bearing restrictions after LLP fixation [9, 10, 20].

Immediate and unrestricted weight-bearing following LLP fixation of distal femoral fractures has been examined by two previous studies [5, 23]. Poole et al. [5] reported that immediate unrestricted weight-bearing after LLP fixation was not associated with failure of fixation, in a study of 122 patients. However, this was not limited to older patients and included predominantly native knees, as opposed to periprosthetic fractures. In a study of 135 patients over 60 years of age with extra-articular native distal femoral fractures, Lieder et al. [23] found no difference in major adverse events (including fixation failure and infection) within 6 months of fixation between patients prescribed immediate WBAT, compared with patients prescribed initial touchdown weight-bearing, with reoperation rates of 11% and 19%, respectively. Although this study included all types of fixation, rather than LLP only, and was not limited to periprosthetic fractures, it seems reasonable to consider PDFFs as similar to extra-articular distal femoral fractures: the femoral component must be well fixed to consider fixation rather than revision and, by definition, there cannot be intra-articular extension. The current study supports this: unrestricted weight-bearing after LLP fixation of PDFFs appears safe, provides adequate reduction and adequate initial fixation is achieved.

Most studies limited to PDFFs have employed weight-bearing restrictions of duration 6–12 weeks [9, 10, 20]. One previous study of 52 patients allowing immediate unrestricted weight-bearing after minimally invasive LLP fixation reported a favourable reoperation rate of 5/54 (9%) [24]. Although this previous study did not include a RWB group for comparison, this reoperation figure compares favourably with both that in the current study and those in studies where weight-bearing restrictions were applied: Lotzien et al. [9] *n* = 45, reoperation rate 22%; Hoellwarth et al. [10], *n* = 87, reoperation rate 10%; Ruder et al. [20], *n* = 35, reoperation rate 6%. Previous studies have demonstrated that imposing weight-bearing restrictions on similar patients is associated with significant morbidity [19, 31]. In the absence of an apparent benefit of weight-bearing restrictions, the current study suggests that weight-bearing should be permitted following LLP fixation of PDFFs.

Medial comminution and non-anatomic reduction were identified as independent predictors of fixation failure and reoperation. This is consistent with previous studies, where medial comminution has been associated with both early failure and non-union [19, 32]. Adequate fixation should be employed to facilitate immediate weight-bearing. This may not always be achievable, for example where femoral stems are present in interprosthetic fractures, but should be the aim. LLP construct stability can be improved by augmentation with additional metalwork, such as dual plating [33] or using a nail-plate construct [33], especially when medial comminution is present. In the current study, the primary LLP fixation device was often augmented with cables and/or dual plating at the discretion of the operating surgeon. Eight surgeons were included; although the influence of the operating surgeon is a source of potential bias in terms of weight-bearing restrictions, the number of surgeons involved may strengthen the generalizability of the results – despite different surgeons and different techniques, the weight-bearing status did not appear to affect fixation failures and the reoperation rate.

In terms of loading the fixation construct, body weight may be relevant, especially in the context of other risk factors for mechanical failure such as medial comminution, suboptimal reductions or more distal fractures. Weight and BMI were not included as covariates in the Cox regression analysis, as these data were absent for six patients and led to a model that was not statistically significant. Although weight-bearing was allowed in the WBAT group, we cannot be sure how much weight patients were actively bearing postoperatively, especially in the early postoperative period (or indeed the time taken until full weight-bearing occurred). However, by not placing any restrictions on patients, patients limit their own weight-bearing to a level that is comfortable and, provided that they have normal sensation, we would suggest that this is safe. This approach appears to be supported by the findings of the current study. The unintended consequence of placing weight-bearing restrictions on frail older patients, who are often cognitively impaired, is that they cannot manage it and so, instead of mobilizing and weight-bearing as they are able, they are not mobilized at all rather than breach their prescription.

The current study has a number of limitations, including its retrospective nature. The study was not randomized and there is substantial potential for selection bias: cases with poorer bone quality and less robust fixation may be more likely to have been prescribed RWB but also be more likely to fail; however, other than the frequency of interprosthetic fractures, no significant difference in baseline characteristics was observed between the two groups. In addition, the patient groups were not of equal size and patient-reported outcomes and quality of life were not assessed. Although increasing in incidence [3–5], PDFFs are not common; it is therefore difficult to power a study adequately from a single centre, and multicentre studies would be desirable. However, it has also been demonstrated that, even with a multicentre design, it may not be feasible to undertake prospective comparative studies for this fracture type within the United Kingdom [34], due to fracture incidence and patient complexity. Although this study may be underpowered, it confirms that, when PDFFs are managed with LLP fixation, unrestricted weight-bearing does not appear to cause catastrophic failure of fixation in these frail older patients. This represents one of the largest series of PDFFs treated with LLPs in the literature. Minimum follow-up was 1 year, which is similar to other studies of PDFFs [10, 20]. This time period can be expected to cover fracture-related complications such as fixation failures and non-union [33], although it is too short to comment on potential longer-term component loosening.

## Conclusion

This study of 43 consecutive patients undergoing fixation of PDFFs with LLPs at a single institution did not demonstrate an increased rate of reoperation or fixation failure when early unrestricted weight-bearing was permitted. Consistent with previous studies, almost one in five patients required reoperation, and this was independently predicted by medial comminution and non-anatomic reduction [22]. When medial comminution is present, LLPs should be augmented with additional fixation, for example dual plating or an intramedullary nail, to provide a construct that is rigid enough to facilitate unrestricted weight-bearing in these typically frail and older patients. Where the surgeon is confident in the fixation construct, weight-bearing restrictions appear unnecessary and, given their potential associated morbidity, should be avoided where possible.

## Data Availability

The datasets during and/or analysed during the current study are available from the corresponding author on reasonable request.
